# Supplementary material for: SHAPS-C: the Snaith-Hamilton pleasure scale modified for clinician administration
Source: PeerJ. 2014 Jun 17;2:e429. doi: 10.7717/peerj.429 (PMC4081294; doi:10.7717/peerj.429)
Supplement: Supplemental Information — A 14 item scale modified based on the original self administered SHAPS tool. This measure permits the assessment of anhedonia via clinician administered questions. [file peerj-02-429-s001.docx]

**Supplemental Information**

***SHAPS-C***

***The Snaith-Hamilton Pleasure Scale- Clinician Administered***

The purpose of this assessment is to evaluate the ability to enjoy/experience pleasure during the past week [or another time frame].

- First, determine if any pleasure was experienced in the given area and then rate the reported level of pleasure.
- In the absence of the actual experience, assess the patient’s anticipation of pleasure. For example: “If you had the opportunity to eat your favorite food, do you think you would enjoy it?” Decreased energy and diminished concentration can commonly interfere with engaging in some of the areas under question in this assessment. Differentiate such symptoms from anhedonia. For example: “if you were not feeling so tired and could go for a walk to your favorite beach do you think you would enjoy the walk?” or “if you could concentrate, do you think you would enjoy reading?”
- It is possible that the area under question has never been pleasurable for the individual, regardless of their mood state. In such cases, assess changes that might have occurred. For example: “I have never enjoyed taking showers but when I am depressed I really cannot get myself to do it, even the idea of taking a shower is unpleasant.” This response would be scored 4. However if the person said “I have never enjoyed taking showers and I still feel the same about it, I do it out of habit”. This response would be scored 2.

1. **Pleasure/enjoyment from favorite radio/TV program**: (Questions to consider: Have you had any pleasure or enjoyment from radio/TV programs or similar activities during the past [time frame]? What are your favorite programs? Have you watched/listened to them? [if not] Do you think you would enjoy them if you did? Is your experience of pleasure in this area similar or different from usual?):

4. No pleasure

3. Some pleasure

2. Average/usual pleasure

1. Lots of pleasure

1. **Pleasure/enjoyment from family or close friends**: (Questions to consider: Who are some of the people you usually spend time with? Family or friends? Have you spent any time with them during the past [time frame]? Talked on the phone? Any contact? Did you enjoy such contacts? Do you think you would enjoy them if you did? Is your experience of pleasure in this area similar or different from usual?):

4. No pleasure

3. Some pleasure

2. Average/usual pleasure

1. Lots of pleasure

1. **Pleasure/enjoyment from hobbies or pastimes:** (Questions to consider: What are some of your hobbies/pastime activities? Have you done any during the past [time frame]? Did you enjoy them? Do you think you would enjoy them if you did? Is your experience of pleasure in this area similar or different from usual?):

4. No pleasure

3. Some pleasure

2. Average/usual pleasure

1. Lots of pleasure

1. **Pleasure/enjoyment from a favorite meal/food:** (Questions to consider: What are your favorite foods? Have you had any during the past [time frame]? Did you enjoy yourself? If not, do you think you would enjoy them if you did? Is your experience of pleasure in this area similar or different from usual?):

4. No pleasure

3. Some pleasure

2. Average/usual pleasure

1. Lots of pleasure

1. **Pleasure/enjoyment from a warm bath or refreshing shower**: (Questions to consider: Do you usually enjoy a warm bath or a refreshing shower? Have you taken any this past [time frame]? Did you enjoy it? If not, would you if you had? Is your experience of pleasure in this area similar or different from usual?):

4. No pleasure

3. Some pleasure

2. Average/usual pleasure

1. Lots of pleasure

1. **Pleasure/enjoyment from scent of flowers, smell of a fresh sea breeze, or freshly baked bread**: (Questions to consider: What are your favorite flowers? What kind of perfumes/scents do you like? Baked bread? What else? Have you had any of these experiences during the past [time frame]? Would it give you any pleasure if you had? Is your experience of pleasure in this area similar or different from usual?):

4. No pleasure

3. Some pleasure

2. Average/usual pleasure

1. Lots of pleasure

1. **Pleasure/enjoyment from seeing other people’s smiling faces:** (Questions to consider: I assume you have come in contact with several people during the past [time frame]. How does it feel when they smile? How about smiling children? If not, would you if you had? Is your experience of pleasure in this area similar or different from usual?):

4. No pleasure

3. Some pleasure

2. Average/usual pleasure

1. Lots of pleasure

1. **Pleasure/enjoyment from looking handsome/beautiful/sharp after an effort to look nice:** (Questions to consider: have you made any effort to look nice this past [time frame]? Did it give you any experience of enjoyment/pleasure? If you had the opportunity to make yourself look nice, would you enjoy it? Is your experience of pleasure in this area similar or different from usual?):

4. No pleasure

3. Some pleasure

2. Average/usual pleasure

1. Lots of pleasure

1. **Pleasure/enjoyment from reading a book, magazine, or newspaper**: (Questions to consider: Have your read any books in the past [time frame]? Do you generally like to read or look at magazines? Newspaper? Similar activities? If concentration were not a problem, would you enjoy reading a book? Looking at a magazine? Is your experience of pleasure in this area similar or different from usual?):

4. No pleasure

3. Some pleasure

2. Average/usual pleasure

1. Lots of pleasure

1. **Pleasure/enjoyment from a favorite beverage**: (Questions to consider: What is your favorite drink? Coffee? Tea? Water? Other? Have you had any in the past [time frame]? Did you enjoy it? Would you if you had any? Is your experience of pleasure in this area similar or different from usual?):

4. No pleasure

3. Some pleasure

2. Average/usual pleasure

1. Lots of pleasure

1. **Pleasure/enjoyment from small things:** (Questions to consider: What are some of the small things you have enjoyed? Taking note of something small? A bright sunny day? A star on your child’s homework? Have you experienced any such small pleasures in the last [time frame]? What other small pleasures do you remember from the past? Would you enjoy them now? Is your experience of pleasure in this area similar or different from usual?):

4. No pleasure

3. Some pleasure

2. Average/usual pleasure

1. Lots of pleasure

1. **Pleasure/enjoyment from a beautiful landscape or view:** (Questions to consider: Do you like nature? What are some of your favorites? What kind of landscape or view? Have you seen any in the past [time frame]? Did you enjoy it? Would you enjoy if you had seen /experienced any? Is your experience of pleasure in this area similar or different from usual? ):

4. No pleasure

3. Some pleasure

2. Average/usual pleasure

1. Lots of pleasure

1. **Pleasure/enjoyment from helping others**: (Questions to consider: Do you usually like to help people? Have you helped anyone during the past [time frame]? What was that like? Would it make you feel good if you had the opportunity to help someone? Is your experience of pleasure in this area similar or different from usual?):

4. No pleasure

3. Some pleasure

2. Average/usual pleasure

1. Lots of pleasure

1. **Pleasure/enjoyment from receiving praise from others** (Questions to consider: have you received any complements/praise? What kind of complements/recognition do you usually enjoy getting? What was that like? How do you think you would feel if you received complements/praise/recognition in the past [time frame]? Is your experience of pleasure in this area similar or different from usual?):

4. No pleasure

3. Some pleasure

2. Average/usual pleasure

1. Lots of pleasure

Total score (range: 14-56):____
